# Does Adding a Cardia Biopsy Improve Gastric Intestinal Metaplasia Detection Rate by the Sydney System Protocol?

**DOI:** 10.34172/aim.2022.63

**Published:** 2022-06-01

**Authors:** Ghazaleh Soltani, Bahar Saberzadeh- Ardestani, Masoud Sotoudeh, Siavosh Naseri -Moghaddam, Mohammad Hossein Derakhshan, Hiva Saffar, Amir Kasaeian, MohammadReza Chavoshi, Alireza Sima

**Affiliations:** ^1^Digestive Disease Research Center, Digestive Disease Research Institute, Tehran University of Medical Sciences, Tehran, Iran; ^2^Institute of Infection, Immunity & Inflammation, University of Glasgow, Glasgow, United Kingdom; ^3^Department of Pathology, Shariati Hospital, Tehran University of Medical Sciences, Tehran, Iran; ^4^Hematology, Oncology and Stem Cell Transplantation Research Center, Tehran University of Medical Sciences, Tehran, Iran, Shariati Hospital, Tehran University of Medical; ^5^Department of Radiology, Shariati Hospital, Tehran University of Medical Sciences, Tehran, Iran

**Keywords:** Cardia, Gastric biopsy, Intestinal metaplasia, Precancerous gastric lesion, Sydney system

## Abstract

**Background:**

The Sydney system offers a standard biopsy protocol for detection and follow-up of gastric preneoplastic lesions such as intestinal metaplasia (IM). The highest frequency of cardia-type gastric adenocarcinoma (GA) in Iran has been documented in the north-western part of the country. This study aims to investigate the effect of the addition of mucosal biopsies of gastric cardia to the standard Sydney protocol on the rate of detection of IM in the asymptomatic residents of this high-risk region for proximal gastric cancer.

**Methods:**

A retrospective new analysis was performed on the previous data obtained in cross-sectional endoscopic screening in 2000 as well as a biopsy study of 508 asymptomatic volunteer residents in Meshkinshahr district, Ardabil province. The screening study was conducted in a group of residents aged 40 years and older who did not have any previous GI or hemodynamic problems.

**Results:**

Intestinal metaplasia at the Sydney protocol sampling sites was detected in 107 samples belonging to 76 of the 508 (14.99%) volunteers. Twenty-one patients had IM at the cardia. Of these, five patients had IM-cardia (IM only at the cardia). Therefore, adding a cardia biopsy to the set of biopsies diagnosed five more IM cases which were not diagnosed on the standard Sydney protocol (*P*=0.062).

**Conclusion:**

The addition of a biopsy from the cardia to the Sydney protocol biopsy set does not seem to improve the frequency of detection of IM in the residents of this high-risk geographic area for proximal gastric carcinoma.

## Introduction

 Gastric adenocarcinoma (GA) is the fifth most frequent cancer and the third leading cause of cancer-related death worldwide, and the leading cause of cancer-related death in Iran.^1,2^ It may arise either from the cardia (CGA) or from the more distal parts – the non‐cardia adenocarcinomas (NCGA).^2-5^ According to recent epidemiological data, CGA is more prevalent in industrialized nations,^6,7^ while NCGA is seen more often in developing countries.^7^ While intestinal metaplasia (IM) is considered to be the precursor tissue for GC in both anatomic sites, the carcinogenetic mechanisms and possible etiologies differ, with *Helicobacter pylori* being the main culprit in NCGA.^8-14^ The Sydney System, first introduced in 1990, offers a comprehensive protocol biopsy plan from standard locations of gastric mucosa and a visual scale to ensure more accurate and reproducible documentation of various histological findings.^15^ In 2001, it was revised by adding one sample from the incisura angularis to the previous four-biopsy set to increase the yield of *H. pylori* infection diagnosis.^16,17^ Further studies, however, showed that IM or other precancerous lesions could be underdiagnosed in some patients; even after the addition of the incisura angularis biopsy to the standard four-biopsy sample, some of the precancerous lesions located elsewhere in the stomach could naturally be missed.^11,17^

 The recommendations of the Sydney protocol in conducting upper gastrointestinal (GI) endoscopy and biopsy are proven to be effective and efficient in the detection of IM and other precancerous lesions in the gastric mucosa.^10,18^

 National surveillance studies conducted by the Iranian Ministry of Health and provincial cancer registry data show that GA is the most fatal cancer in Iran, especially in the Ardabil province.^12,14^ According to the available studies, CGA is more prevalent in north and north-western Iran, including the Ardabil province.^14,18^ To characterize the precancerous lesions among asymptomatic individuals, an endoscopic study was performed on volunteers in the region two decades ago.^18^ The investigators had taken protocol biopsies from the cardia, incisura, lesser and greater curves of the body and antrum in addition to any suspicious lesion.^10,13,19,20^

 With this background and the knowledge of the high incidence of CGA in the Ardabil province, we used the available pathology data from the Ardabil study to see if taking a cardia biopsy may add to the IM detection rate in healthy volunteers.^21,22^

## Materials and Methods

###  Study Design and Participants

####  Study Population

**Figure 1:**
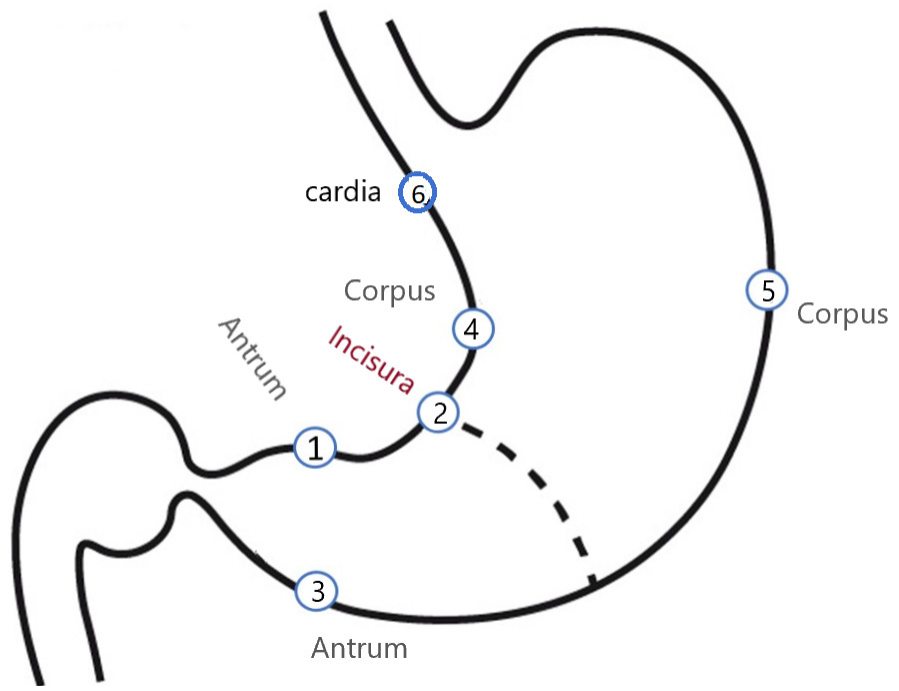
Biopsy Mapping. Biopsy Sites: **1:** Antrim: Prepyloric, 2 cm of Pylorus, **2:** Distal antrum, Lesser Curvature, **3:** Mid antrum, Greater Curvature, **4:** Mid Body(corpus), Lesser Curvature, **5:** Mid corpus, Greater Curvature, **6:** Cardia: Just below the Z line. In the presence of any gross masses, a biopsy was taken to investigate further.

 We used the histopathology data obtained from slides of the aforementioned study in Meshkinshahr, Ardabil.^18^ Briefly, in that study, 1,105 households were canvassed, and residents aged 40 years and older without upper GI symptoms were invited to participate. Those consenting volunteers without a history of significant benign or malignant upper GI disease, recent gastric surgery, coagulopathy (INR > 1.5 or platelet count < 150 × 10^3^), and hemodynamic instability underwent upper gastrointestinal endoscopy and protocol biopsies were taken from cardia, incisura, and lesser and greater curves of the antrum and body. Figure 1 demonstrates the map of gastric biopsy sites.

 Of the 508 individuals enrolled in the Meshkinshahr study, 409 had a complete set of gastric biopsies (including the cardia). In 15 individuals, 7 biopsy samples were taken; the additional biopsy was taken due to observation of a suspicious mass.

 All the biopsies were fixed in 10% buffered formalin, processed, embedded in paraffin blocks, sectioned, and stained by hematoxylin-eosin (H&E) Giemsa methods following standard procedures. Two pathologists specializing in GI pathology reviewed the gastric biopsy slides, each going through 50% of the slides. They reviewed the first 20 slide sets in joint sessions to become acquainted with and establish a preliminary agreement on the histological criteria and definitions presented in the Sydney system. Then, they recorded the histological changes, including pattern and degree of inflammation, presence of *H. pylori*, IM, and mucosal atrophy according to the criteria and visual scales provided in the Sydney protocol. Both pathologists reviewed a randomly chosen sample of 10% of the slides for the purpose of accuracy and reproducibility assurance. All problems and borderline cases were also reviewed by both pathologists, and discrepancies were resolved in joint sessions. Numerical values were assigned to the type and degree of histological findings. If IM was present on any of the antral, corpus, or incisura biopsies in a given case, that case was diagnosed as having IM. Then, the cardia biopsy readings were assessed, and the number of cases with IM was recorded. The cases with IM at the cardia but not on biopsies from other parts of the stomach were designated as IM-cardia.

###  Statistical Analysis

 McNemar’s test was used to compare percentage of positive results detected by each protocol. Besides, we used kappa (κ) coefficient as an interobserver agreement that points to the degree of agreement beyond what would be expected by chance alone and typically ranges from -1 to + 1, where a greater value indicates better reliability such that, for example, a kappa of 0.80 to 1.00 indicates excellent agreement.^23^ *P *values < 0.05 reflected statistically significant agreement. STATA, version 16 for Windows (StataCorp. 2019. Stata Statistical Software: Release 16. College Station, TX: StataCorp LP) was used for the statistical analysis.

## Results

 Three thousand and eighty gastric biopsy samples were obtained from 508 participants. A complete set of six-biopsy samples was obtained from the six regions as shown in Figure 1 and slides were prepared for evaluation. An extra biopsy was obtained from 32 participants due to observation of a suspicious gastric lesion during upper endoscopy. General histopathological findings are shown in Table 1. Of the 508 participants, 7 participants had no significant histopathological changes in any of the biopsy sites. In the remaining 500 participants, 76 participants had IM on at least one site (excluding cardia), with antrum being the most common site for IM (38 participants, 35.5%) (Table 2). Naturally, there are overlaps in the detection of various pathological changes in any slide set belonging to a participant. Dysplasia was detected in one and adenocarcinoma in another participant (each 0.02%) (Table 3).

**Table 1:** Frequency of Histopathological Changes

**Histological Changes**	**No**. ** (%)**
Normal (all samples)	7 (1.38)
Active chronic gastritis	216 (42.69)
Chronic gastritis	497 (97.83)
Atrophy	33 (6.50)
Intestinal Metaplasia	76 (14.99)
Reactive atypia	214 (42.21)
Dysplasia	1 (0.20)
Adenocarcinoma	1 (0.20)

**Table 2:** Prevalence of Distribution of *Helicobacter pylori* Based on Biopsy Site

**Biopsy Region**	**No**. ** (%)**
Antrum: Prepyloric, 2 cm of pylorus	336 (67.20)
Antrum: Distal antrum, lesser curvature	338 (67.33)
Antrum: Mid antrum, greater curvature	305 (61.12)
Body: Mid body, lesser curvature	288 (57.95)
Body: Mid body, greater curvature	261 (51.99)
Cardia: Just below the Z line	245 (49.80)

**Table 3:** Frequency of Histopathological Changes Based on Biopsy Site

**Biopsy Site, No. (%)**	***H. pylori***	**IM**	**Glandular Dysplasia**	**Adenocarcinoma**	**Dysplasia or Adenocarcinoma**
1	336 (67.20)	38 (7.50)	0	0	0
2	338 (67.33)	43 (8.48)	0	1 (0.20)	1 (0.20)
3	305 (61.12)	33 (6.51)	0	0	0
4	288 (57.95)	33 (6.51)	1 (0.20)	0	1 (0.20)
5	261 (51.99)	14 (2.76)	0	0	0
6	245 (49.80)	21 (4.14)	0	0	0
1 or 2	374 (73.77)	67 (13.21)	0	1 (0.20)	1 (0.20)
1 or 2 or 3	388 (76.53)	86 (16.96)	0	1 (0.20)	1 (0.20)
4 or 5	327 (64.50)	42 (8.28)	1 (0.20)	0	1 (0.20)

IM, intestinal metaplasia. 1, antrum in 2 cm distance from the pyloric canal; 2, Antrum Lesser Curvature; 3, Antrum Greater Curvature; 4, Corpus Lesser curvature; 5, Corpus Greater Curvature; 6, cardia just below the Z line; IM, Intestinal Metaplasia.

 Comparing the set of Sydney protocol biopsies with cardia biopsies in terms of IM diagnosis was not statistically significant, although considered significant at the 0.10 level of significance (*P* = 0.063)

Table 4 illustrates the consistency in the detection of disease based on the two different protocols. The McNemar’s test *P *values and the estimated kappa coefficient, the *P *value of the kappa tests and the limits of the 95% confidence interval for the Kappa coefficients are presented for each biopsy area in this table. The results of both tests were consistent and confirmed each other. The findings disclosed that the interobserver agreement between the two protocols were excellent for all five areas such that for three of them, including glandular dysplasia, adenocarcinoma, and dysplasia or cancer, agreement was complete. The findings also showed that the addition of cardia biopsies (the study protocol) did not significantly increase the detection rate of IM in comparison with the standard Sydney protocol biopsies alone [McNemar’s test *P* = 0.0625, kappa = 97.09 (*P* < 0.0001)].

**Table 4:** Comparison of Consistency in Detecting Histopathological Changes by Sydney Protocol vs. Study Protocol Regarding Cardia Biopsy

			**Sydney Biopsy plus Additional Cardia Biopsy**			**McNemar’s Test ** ***P*** ** Value**	**Cohen's Kappa** **(** ***P*** ** Value)**
			**Negative**	**Positive**	**Total**		
Sydney biopsy alone	HP	Negative	99 (19.49%)	2 (0.39%)	101 (19.88%)	0.50	98.75 (< 0.0001)
		Positive	0 (0.00%)	407 (80.12%)	407 (80.12%)		
		Total	99 (19.49%)	408 (80.51%)	508 (100%)		
	IM	Negative	396 (77.95%)	5 (0.98%)	401 (78.94%)	0.0625	97.09 (< 0.0001)
		Positive	0 (0.00%)	107 (21.06%)	107 (21.06%)		
		Total	396 (77.95%)	112 (22.05%)	508 (100%)		
	Glandular dysplasia	Negative	507 (99.8%)	0 (0.00%)	507 (99.8%)	1.000	100 (< 0.0001)
		Positive	0 (0.00%)	1 (0.2%)	1 (0.2%)		
		Total	507 (99.8%)	1 (0.2%)	508 (100%)		
	Adeno carcinoma	Negative	507 (99.8%)	0 (0.00%)	507 (99.8%)	1.000	100 (< 0.0001)
		Positive	0 (0.00%)	1 (0.2%)	1 (0.2%)		
		Total	507 (99.8%)	1 (0.2%)	508 (100%)		
	Dysplasia or cancer	Positive	506 (99.61%)	0 (0.00%)	506 (99.61%)	1.000	100 (< 0.0001)
		Negative	0 (0.00%)	2 (0.39%)	2 (0.39%)		
		Total	506 (99.61%)	2 (0.39%)	508 (100%)		

HP, *Helicobacter pylori*; IM, intestinal metaplasia.

## Discussion

 We studied the significance of adding a cardia biopsy to the Sydney protocol for gastric biopsies using the available samples of 508 asymptomatic people from Meshkinshahr, a high incidence area for GA (both CGC and NCGC). Our data shows that adding the cardia biopsy is able to detect 4.27% more IM than the Sydney protocol (*P* = 0.062). Although the difference was not statically significant, there was a trend toward significance.

 Gastric cardia is a poorly defined area measuring 5–15mm below the gastro-esophageal junction.^24^ Obtaining biopsies exactly from this area may be a difficult task because of the esophagus and stomach’s peristaltic movements and the fact that the gastro-esophageal junction (GEJ) is also seen differently by different endoscopists. The triggering factors and significance of IM at the cardia are also not well established. Some believe it is a consequence of gastro-esophageal reflux disease (GERD), others relate it to *H. pylori* and some to both.^24^ Current data relate it to GERD in Western countries and to *H. pylori* in the East and South Asian countries.^25^ GERD is increasing in Iran and although *H. pylori* infection has decreased significantly, it is still much more common than the West.^26^ A previous study from Iran looking at the GEJ of dyspeptic patients referred for Esophagogastroduodenoscopy (EGD) to a tertiary care GI unit, reported 5% prevalence of IM and 48% prevalence of *H. pylori* at the site.^26^ Western reports indicate the prevalence of IM at the GEJ at 5–18% with less than a third harboring *H. pylori*.^27^

 The clinical behavior of IM at the cardia may be different from that of the adjacent lower esophagus or the more distal parts of the stomach. However, it has been clearly shown that IM at this site has the potential of progression to adenocarcinoma, albeit at a lower rate than the two other mentioned sites.^28^

 To conclude, our data show that adding a cardia biopsy to those proposed by the Sydney system may detect IM not found on other biopsies, but the difference was not statistically significant. Considering the borderline *P* value of 0.063, it could be assumed that if we had studied a larger number of individuals, this figure might have attained statistical significance. As IM at the cardia is prevalent and may be present without IM at other sites, further studies with a larger number of cases are warranted to better delineate the issue.
